# Human Serum Albumin and Human Serum Albumin Nanoparticles as Carriers of 10-(2′-Pyrimidyl)-3,6-diazaphenothiazine: In Vitro Spectroscopic Studies

**DOI:** 10.3390/molecules30020315

**Published:** 2025-01-15

**Authors:** Aleksandra Owczarzy, Karolina Kulig, Beata Morak-Młodawska, Małgorzata Jeleń, Tammam Muhammetoglu, Wojciech Rogóż, Małgorzata Maciążek-Jurczyk

**Affiliations:** 1 Department of Physical Pharmacy, Faculty of Pharmaceutical Sciences in Sosnowiec, Medical University of Silesia, 40-055 Katowice, Poland; aowczarzy@sum.edu.pl (A.O.); kkulig@sum.edu.pl (K.K.); wrogoz@sum.edu.pl (W.R.); 2 Department of Organic Chemistry, Faculty of Pharmaceutical Sciences in Sosnowiec, Medical University of Silesia, 40-005 Katowice, Poland; bmlodawska@sum.edu.pl (B.M.-M.); manowak@sum.edu.pl (M.J.); 3 Pavia University, 27100 Pavia, Italy; t.muhammetoglu98@gmail.com

**Keywords:** human serum albumin, 10-(2′-pyrimidyl)-3,6-diazafenothiazine, nanoparticles, spectroscopy

## Abstract

Human serum albumin (HSA) plays a fundamental role in the human body, including the transport of exogenous and endogenous substances. HSA is also a biopolymer with a great medical and pharmaceutical potential. Due to nontoxicity and biocompatibility, this protein can be used as a nanocarrier. 10-(2′-Pyrimidyl)-3,6-diazaphenothiazine (10-Pyr-3,6-DAPT) is a phenothiazine showing high anticancer potential in vitro against glioma, melanoma and breast cancer cells. Additionally, this compound is characterized by selectivity of action towards MCF-7 breast cancer and has low cytotoxicity towards normal cells. Considering the promising pharmacological potential of this compound and using spectroscopic techniques, HSA and human serum albumin nanoparticles (HSA-NP) were tested as carriers of this molecule. Based on the obtained data and the appropriate mathematical models (Stern-Volmer and Klotz models), it can be concluded that 10-Pyr-3,6-DAPT probably forms a weak (K_a_ = (5.24 ± 0.57) × 10^4^ and K_a_ = (4.67 ± 0.59) × 10^4^) for excitation wavelengths λ_ex_ 275 nm and λ_ex_ 295 nm, respectively) static complex (k_q_ > 10^10^) with HSA (at Sudlow site II (subdomain IIIA), and the phenomenon of it having both strong therapeutic and toxic effects is possible. High encapsulation efficiency of 10-Pyr-3,6-DAPT into the HSA-NPs was obtained, and the changes in albumin secondary structure due to the presence of 10-Pyr-3,6-DAPT were registered. Based on the data presented, it can be concluded that due to the high toxic effects of 10-Pyr-3,6-DAPT, a better carrier may be HSA-NPs.

## 1. Introduction

Human serum albumin (HSA) is the most abundant protein in the bloodstream. HSA is synthesized by liver cells (hepatocytes). It plays numerous functions in the human body, including the transport of exogenous and endogenous substances. HSA actively participates in the maintenance of appropriate oncotic pressure, or pH. This protein is also a crucial indicator of inflammation throughout the body [1,2]. HSA is heart-shaped, with dimensions of 80 × 80 × 30 Å and molar mass of molecule approximately 66.5 kDa. The secondary structure of HSA is dominated by α-helix (around 67%) without β-sheets while the tertiary structure consists of three homologous domains (I–III) which are divided into two subdomains (A, B) [3,4]. In the molecular structure of HSA, according to Sudlow’s nomenclature, two binding sites (I, II), in subdomain IIA and IIIA, can be distinguished. These two binding sites play a key role in drug distribution in the human bloodstream [1,3,4].

**Figure 1:**
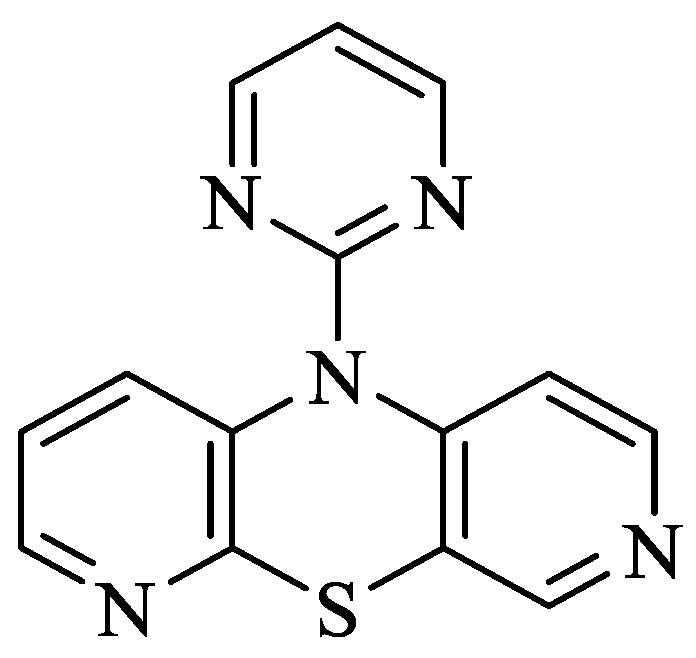
Structure of 10-(2′-pyrimidyl)-3,6-diazafenothiazine [8].

Phenothiazines (PTZ) are heterocyclic compounds having a 1,4-thiazine ring in their structure [5]. These compounds are known primarily for their antipsychotic properties, which allowed their use in psychiatry in the treatment of schizophrenia and other manic states [6]. Additionally, they have other valuable biological properties, e.g., antibacterial, antiviral and anticancer properties, which are extremely useful in many areas of drug discovery [7,8,9]. Dipyridothiazines are a group of modified phenothiazines, having in their structure two pyridine rings instead of two benzene rings [10,11]. They are systems with valuable anticancer, immunosuppressive and antioxidant potential [10]. In the group of these compounds, 10-(2′-pyrimidyl)-3,6-diazaphenothiazine (10-Pyr-3,6-DAPT) occupies a special place; it is characterized by high anticancer activity determined in vitro and low cytotoxicity. It inhibits the growth of SNB-19 glioma, C-32 melanoma and MCF-7 breast cancer cell lines [12,13]. More importantly, it showed high selectivity towards the MCF-7 breast cancer cell line, having an IC_50_ = 0.78 μg/mL. Analysis of the mechanism of anticancer activity showed that this molecule has the ability to activate the mitochondrial apoptosis pathway, influencing the regulation of the following genes: *H3* (proliferation marker), *TP53* and *CDKN1A* (cell cycle regulating genes), *BCL-2* and *BAX* (apoptosis pathway markers) [12]. This molecule showed a significant increase during gene expression studies *CDKN1A* copies in MCF-7 and SNB-19 cells, suggesting this possibility involved in cell cycle arrest and apoptosis. Figure 1 shows the structure of 10-(2′-pyrimidyl)-3,6-diazaphenothiazine).

Due to the fact that only free fraction of the drug (not bound with carrier protein) can cause both therapeutic and toxic effects, the determination of free and bound fractions concentration is very important. Furthermore, in a complementary way, the drug concentration in the target tissue is related to the concentration of unbound drug in human plasma [14,15,16].

Nanotechnology is the production of materials at a nanoscale level. Based on the dimension, there are 0D, 1D, 2D or 3D nanomaterials, and depending on the shape, they can be divided into several groups [17]. Nowadays, polymeric nanoparticles are commonly used for medical and pharmaceutical applications as a drug carries and diagnostic agents [18]. One of the main advantage of nanocarriers is the ability to increase the solubility of drugs due to encapsulation into a polymeric core. Moreover, drug encapsulation enhances drug permeability through different route of its administration and prevents the nonspecific binding to healthy tissues. This can be achieved due to targeting to the reticuloendothelial system, inflammation sites or specific receptors on the surface of cells [19]. Due to the long half-life of albumin nanoparticles, as well as its biodegradability and biocompatibility, it is a widely used polymer in the study of drug delivery systems. In addition, its ability to bind to gp60 allows it to penetrate cancer cells more efficiently than nanocarriers made from other polymers [19].

So far, DAPT has not been encapsulated in nanoparticles, and studies of its interaction with HSA have not been performed. Taking into account the above reports, the main aim of this project was to investigate whether both human serum albumin and human serum albumin nanoparticles could be treated as carriers of a newly synthesized substance with potential anticancer activity.

## 2. Results

### 2.1. Study of HSA as a Carrier of 10-Pyr-3,6-DAPT

**Figure 2:**
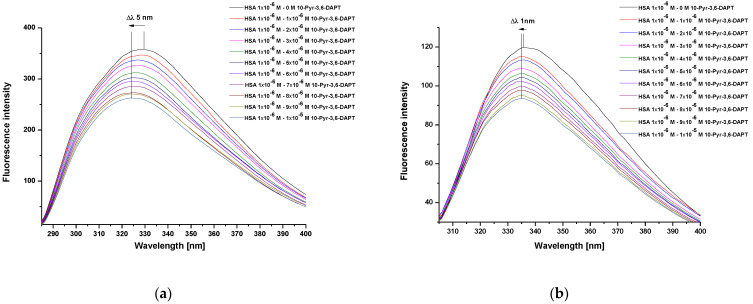
Steady state fluorescence spectra of HSA (1 × 10^−6^ mol·L^−1^) in the absence and presence of 10-Pyr-3,6-DAPT (1 × 10^−6^ mol·L^−1^–1 × 10^−5^ mol·L^−1^) at λ_ex_ 275 nm (**a**) and λ_ex_ 295 nm (**b**) excitation wavelengths.

Steady state fluorescence spectra of human serum albumin (HSA) at 1 × 10^−6^ mol·L^−1^ concentration, both in the absence and presence of 10-(2′-pyrimidyl)-3,6-diazaphenothiazine (10-Pyr-3,6-DAPT), at increasing concentration from 1 × 10^−6^ mol·L^−1^ to 1 × 10^−5^ mol·L^−1^ and at λ_ex_ 275 nm and λ_ex_ 295 nm excitation wavelengths, were recorded (Figure 2a,b).

From the recorded HSA steady state fluorescence spectra, a decrease in the fluorescence intensity of the studied protein in the presence of 10-Pyr-3,6-DAPT has been observed (Figure 2a,b). In addition, a hypsochromic shift (blue shift) of maximum fluorescence intensity, both at λ_ex_ 275 nm (Δλ 5 nm) and λ_ex_ 295 nm (Δλ 1 nm) excitation wavelengths, has been recorded. To confirm this shortwave spectrum shift, especially at λ_ex_ 295 nm excitation wavelength, a spectral parameter A (based on Equation (2)) and a full width at half maximum (FWHM) have been calculated and collected in Table 1.

Based on the data collected in Table 1, a decrease in the values of spectral parameter A and FWHM with the increase of [10-Pyr-3,6-DAPT]:[HSA] molar ratio has been observed.

**Figure 3:**
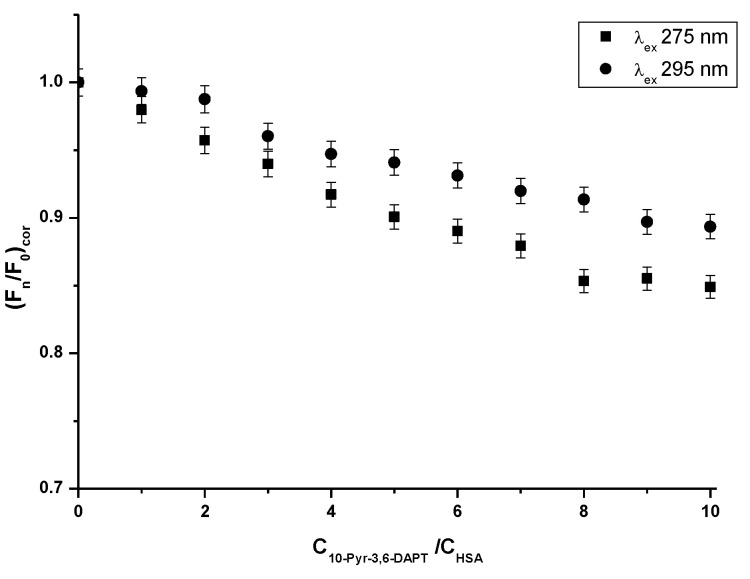
Fluorescence quenching curves of HSA (1 × 10^−6^ mol·L^−1^) in the absence and presence of 10-Pyr-3,6-DAPT (1 × 10^−6^ mol·L^−1^–1 × 10^−5^ mol·L^−1^) at λ_ex_ 275 nm and λ_ex_ 295 nm excitation wavelengths.

From the steady-state fluorescence spectra data, the fluorescence quenching curves of HSA in the presence of 10-Pyr-3,6-DAPT (1 × 10^−6^ mol·L^−1^–1 × 10^−5^ mol·L^−1^) have been plotted and presented in Figure 3.

Based on the steady state fluorescence spectra (Figure 2) and the fluorescence quenching curves presented in Figure 3, a decrease in the fluorescence intensity of HSA in the presence of 10-Pyr-3,6-DAPT, at both excitation wavelengths (λ_ex_ 275 nm and λ_ex_ 295 nm), has been noticed. Moreover, it can be seen that the fluorescence quenching curves of HSA (λ_ex_ 275 nm and λ_ex_ 295 nm) do not overlap. In addition, using steady state fluorescence spectra data (Figure 2), the percentage of HSA fluorescence quenching at λ_ex_ 275 nm and λ_ex_ 295 nm excitation wavelengths in the presence of 10-Pyr-3,6-DAPT has been calculated and collected in Table 2.

From the data collected in Table 2, a slight decrease in HSA percentage fluorescence quenching in the presence of 10-Pyr-3,6-DAPT can be observed. However, slightly higher at λ_ex_ 275 nm than at λ_ex_ 295 nm excitation wavelengths.

**Figure 4:**
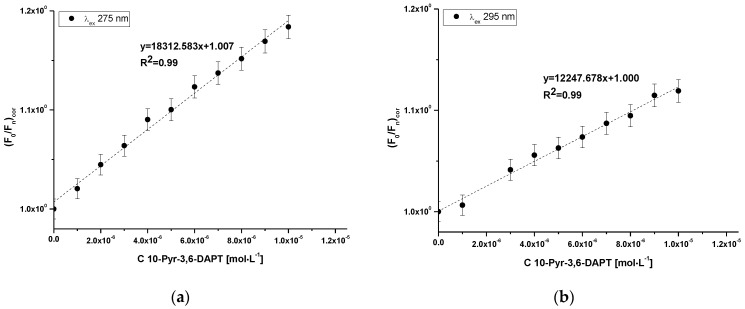
Stern-Volmer plots for 10-Pyr-3,6-DAPT-HSA complex at λ_ex_ 275 nm (**a**) and λ_ex_ 295 nm (**b**) excitation wavelengths; [10-Pyr-3,6-DAPT]:[HSA] 0:1–10:1 molar ratio.

In order to determine the character of albumin fluorescence quenching caused by 10-Pyr-3,6-DAPT, based on the Stern-Volmer equation (Equation (3)), the Stern-Volmer plots have been plotted and presented in Figure 4a,b.

At both λ_ex_ 275 nm and λ_ex_ 295 nm excitation wavelengths, a Stern-Volmer curve has linear course. Accordingly, based on Equation (3), the Stern-Volmer constant (K_S-V_) and the bimolecular quenching constant rate (k_q_) have been calculated and presented in Table 3.

From the data collected in Table 3, it was observed that the values of K_S-V_ as well as k_q_ at both λ_ex_ 275 nm and λ_ex_ 295 nm excitation wavelengths are of the same order.

**Figure 5:**
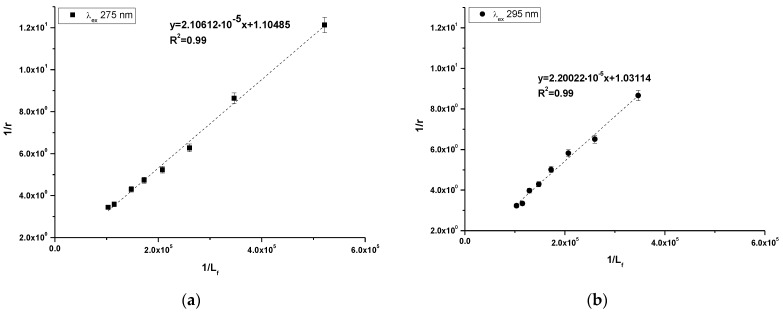
Klotz plots for 10-Pyr-3,6-DAPT-HSA complex at λ_ex_ 275 nm (**a**) and λ_ex_ 295 nm (**b**) excitation wavelengths; [10-Pyr-3,6-DAPT]:[HSA] 0:1–10:1 molar ratio.

Using Equation (4), the Klotz plots have been drawn (Figure 5a,b). Based on Equation (4), the association constant (K_a_ [mol^−1^·L]) and number of binding site classes (n) have been determine and presented in Table 4.

Based on the data collected in Table 4 it can be observed that the obtained values of association constants (K_a_) are in the order of magnitude 10^4^ at both excitation wavelengths. The K_a_ value obtained at λ_ex_ 275 nm excitation wavelength was slightly higher. In addition, the obtained number of binding site classes (n) oscillated around 1 at both λ_ex_ 275 nm and λ_ex_ 295 nm excitation wavelength.

To confirm the high affinity binding sites of 10-Pyr-3,6-DAPT at HSA molecule, the fluorescent markers have been used. Based on the registered steady state fluorescence spectra of the dansylated amino acids, utilizing Equation (5) the percentage of dGlu and dPro displacement from HSA binding site in the presence of 10-Pyr-3,6-DAPT has been calculated and summarized in Table 5.

Based on the data collected in Table 5, the percentage of fluorescence marker displacement in the presence of 10-Pyr-3,6-DAPT was stronger for dPro than for dGlu.

### 2.2. Study of 10-Pyr-3,6-DAPT Encapsulation into HSA-NPs

HSA-NPs and 10-Pyr-3,6-DAPT-HSA-NPs were prepared according to the desolvation method [20,21]. The encapsulation efficiency of the 10-Pyr-3,6-DAPT into the nanoparticles amounted to 99.65 ± 0.05% (n = 3; average ± SD).

### 2.3. Study of HSA and HSA-NPs Secondary Structure

**Figure 6:**
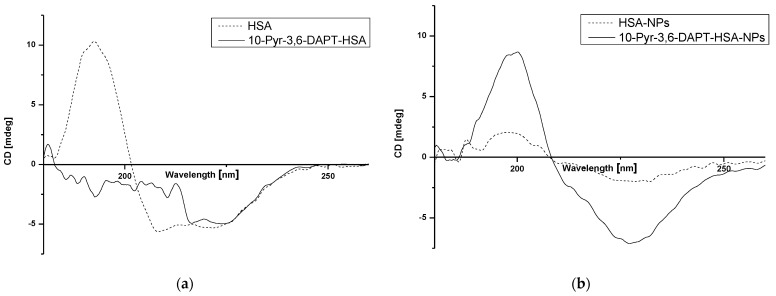
Far UV-CD spectra of HSA and 10-Pyr-3,6-DAPT-HSA (**a**) and HSA-NPs and 10-Pyr-3,6-DAPT-HSA-NPs (**b**).

Circular dichroism (CD) was used to determine changes in secondary structure of human serum albumin (HSA) in the absence and presence of 10-Pyr-3,6-DAPT as well as in human serum albumin nanoparticles (HSA-NPs) after the preparation process. The results are shown in Figure 6a,b.

Based on Figure 6, the changes in intensity as well as shape bands of far UV-CD HSA-NPs spectrum in comparison to HSA spectrum (Figure 6b and Figure 6a, respectively—dotted lines) have been registered. Moreover, in the presence of 10-Pyr-3,6-DAPT, far UV-CD spectra of both HSA and HSA-NPs (Figure 6a and Figure 6b, respectively—solid lines), change significantly. Interestingly, far UV-CD 10-Pyr-3,6-DAPT-HSA-NPs spectrum (Figure 6b—solid line) is very similar to HSA spectrum (Figure 6a—dotted line).

## 3. Discussion

### 3.1. Study of HSA as a Carrier of 10-Pyr-3,6-DAPT

The analysis of changes in the steady state fluorescence spectra intensity of human serum albumin (HSA) in the presence of 10-(2′-pyrimidyl)-3,6-diazafenothiazine (10-Pyr-3,6-DAPT) at increasing concentration made it possible to characterize the intermolecular interaction occurring in the studied system. Due to the fact that both λ_ex_ 275 nm and λ_ex_ 295 nm excitation wavelengths have been used, HSA tyrosyl residues (Tyrs) and one tryptophanyl residue (Trp-214) are mainly responsible for its fluorescence capacity [22]. Based on register steady state fluorescence spectra of HSA in the present of 10-Pyr-3,6-DAPT at increasing concentration (1 × 10^−6^ mol·L^−1^–1 × 10^−5^ mol·L^−1^), a decrease in HSA fluorescence intensity has been observed at both λ_ex_ 275 nm (Figure 2a) and λ_ex_ 295 nm (Figure 2b) excitation wavelengths. This phenomenon is probably related to the direct energy transfer between the protein and studied substances. In addition, the maximum intensity position of the steady-state fluorescence spectra at the wavelength range as well as their shape are characteristic of a defined protein under specific measurement conditions [23]. In the present study, a hypsochromic shift (blue shift) of the maximum fluorescence intensity at both excitation wavelengths has been observed (Δλ 5 nm and Δλ 1 nm at λ_ex_ 275 nm and λ_ex_ 295 nm excitation wavelengths, respectively (Figure 2a,b)). To confirm this phenomenon, especially at λ_ex_ 295 nm excitation wavelength (the shift is within the limits of apparatus error ±1.5 nm), and utilizing Equation (4), the spectral parameter A, which is very sensitive to small changes in the λ_max_ position of the steady state fluorescence spectra, as well as full width at half maximum (FWHM) were calculated. Similarly to the present study, Maciążek-Jurczyk et al. [24] analyzed the effect of oxidative stress on the structure of human albumin as a carrier of a newly synthesized substance with potential anticancer activity and used the spectral parameter A and FWHM to assess changes in the position of the maximum of fluorescence intensity. The authors observed a decrease in albumin fluorescence intensity and values of both spectral parameter A and FWHM. These alterations were attributed to the changes in the tertiary structure of HSA caused by the presence of studied ligand. Based on these conclusions it can be proved that the phenomenon observed in the present study is probably caused by the decrease in exposure of the tryptophanyl and tyrosine residues of albumin to the solvent, and indicates an increase in the hydrophobicity of fluorophores environment, especially the tryptophanyl residue, which is very sensitive to the nature of the immediate environment. Based on this, it can be concluded that 10-Pyr-3,6-DAPT could change the environment around the aromatic amino acids to more hydrophobic, thereby modifying the tertiary structure of HSA.

At λ_ex_ 275 nm excitation wavelength, both tyrosyl residues and one tryptophanyl residue (Trp-214) present in the HSA molecules are excited to fluorescence. In contrast, using λ_ex_ 295 nm excitation wavelength, only Trp-214 excites [25]. Fluorescence quenching curves of 10-Pyr-3,6-DAPT-HSA system at molar ratio [10-Pyr-3,6-DAPT]:[HSA] from 0:1 to 10:1 at both λ_ex_ 275 nm and λ_ex_ 295 nm excitation wavelengths do not overlap (Figure 3). Similar course of fluorescence quenching curves was obtained by Owczarzy et al. [16] in in vitro studies involving the spectroscopic analysis of the interaction between 9-amino-5-alkyl-12(H)-chino[3,4-b][1,4]benzothiazine chloride (Salt3) and major carrier plasma proteins (human serum albumin (HSA) α1 acid glycoprotein (AGP), human γ globulin (HGG) and controlled normal serum (CNS)). For Salt3-AGP complex, the course of fluorescence quenching curves was different. On this basis, the authors concluded that both tyrosyl and tryptophanyl residues present in the AGP molecule were involved in the Salt3-AGP complex formation. Taking into consideration the results obtained in the present work, it could be deduced that both tyrosyl (Tyr-401, Tyr-411, Tyr-497) and Trp-214 residues are involved in the formation of 10-Pyr-3,6-DAPT-HSA complex [26].

From the data collected in Table 2, it can be observed that, in general, the percentage of fluorescence quenching of HSA in the presence of 10-Pyr-3,6-DAPT at increasing concentration is not very high. However, it is slightly stronger at λ_ex_ 275 nm that at λ_ex_ 295 nm excitation wavelength. Maciążek-Jurczyk et al. [27] have demonstrated by spectroscopic analysis of phenylbutazone and ketoprofen binding to HSA that both drugs strongly (in the range of 60–70%) quenched the HSA fluorescence. It is noteworthy that the results obtained in the present study might indicate a weak quenching of HSA fluorescence caused by 10-Pyr-3,6-DAPT. However, the studied substance has a slightly greater ability to quench the fluorescence of tyrosyl residues occurring in the HSA molecule.

In order to determine the character of HSA fluorescence quenching in the presence of 10-Pyr-3,6-DAPT, the Stern-Volmer curves have been plotted (Figure 4a,b), and at both excitation wavelengths a rectilinear course of the curves has been observed. Hence, the nature of HSA fluorescence quenching cannot be precisely identified [28], and based on Equation (3), the Stern-Volmer constants (K_S-V_ [mol^−1^·L]) as well as the bimolecular quenching constant rate (k_q_ [mol^−1^·L·s^−1^] have been calculated (Table 3). The data collected in Table 3 indicate that K_S-V_ and k_q_ values obtained at both excitation wavelengths are of the order of magnitude 10^4^ and 10^12^, respectively. In accordance with Lakowicz theory [29] for dynamic fluorescence quenching, the maximum value of k_q_ in aqueous solution is 10^10^ mol^−1^·L·s^−1^]. In turn, K_S-V_ indicates the distance between the ligand and the excited fluorophore. The higher K_S-V_ value, the closer the ligand is to the excited fluorophore and the stronger the complex formed [22]. As in the present study, Vukic et al. [30] investigated the binding capacity of naphthoquinone derivatives to deoxyribonucleic acid from calf thymus (ctDNA) as well as HSA, and obtained K_S-V_ and k_q_ values of the order of magnitude 10^5^ and 10^13^, respectively. The authors concluded that naphthoquinone derivatives form weak static complexes with ctDNA and HSA. Based on these, it can be concluded that 10-Pyr-3,6-DAPT probably forms a weak static complex with HSA.

In order to assess the stability of the formed complex, based on the Klotz equation (Equation (4), the association constants (K_a_ [mol^−1^·L] and the number of binding site classes (n) have been calculated (Table 4). The obtained values of association constants are in order of magnitude 10^4^, while the number of binding site classes oscillates around 1. In the above described study conducted by Owczarzy et al. [16], the association constants (K_a_) and the number of binding site class (n) values for Salt3-HSA complex were (1.88 ± 0.36) × 10^4^ [mol^−1^·L] and 0.92 ± 0.09 (λ_ex_ 275 nm). In turn, at λ_ex_ 295 nm excitation wavelength, the values were equal to (2.68 ± 0.90) × 10^4^ [mol^−1^·L] and 0.98 ± 0.06, and the authors concluded that Salt3 binds to HSA in one class of binding site and the complex is not stable. Based on the obtained data and data presented by Owczarzy et al., it can be also assumed that 10-Pyr-3,6-DAPT-HSA complex is rather weak and this ligand probably binds to the HSA molecule in one class of binding site. To identify the high affinity binding site of 10-Pyr-3,6-DAPT in the HSA molecule, dansyl-L-glutamine (dGlu) and dansyl-l-proline were used as the fluorescence binding site markers for Sudlow site I (subdomain IIA) and Sudlow site II (subdomain IIIA), respectively [31]. Dansylated amino acids localize to major binding sites in the HSA molecule and show fluorescence activity in complex with the macromolecule, whereas they do not show fluorescence capacity in their unbound form [32]. Based on data collected in Table 5, it can be observed that 10-(2′-pyrimidyl)-3,6-diazafenothiazine displaces fluorescence markers from both binding sites, more strongly from Sudlow site II (56.21 ± 0.56%) than Sudlow site I (39.86 ± 1.09%). Ryan et al. [33], in a study concerning the structural basis of binding of fluorescent, site-specific dansylated amino acids to HSA, confirmed the suitability of using dansylated amino acids as high affinity binding site markers with the HSA molecule. In spectroscopic studies of benzothiazine derivative (Salt1) in terms of the in vitro interaction with selected human plasma proteins, Owczarzy et al. [14] also used dansyl-l-glutamine (dGlu) and dansyl-l-proline (dPro) as high affinity binding site markers. The authors of the paper proved that Salt1 displaces fluorescent markers from their binding sites in HSA structure and probably binds at HSA both in IIA and IIIA subdomains. Based on this, it can be concluded that Sudlow site II (subdomain IIIA) could be, in HSA, the main binding site of 10-Pyr-3,6-DAPT. However, the subdomain IIA might also be involved in the binding of the studied ligand.

### 3.2. Study of 10-Pyr-3,6-DAPT Encapsulation into HSA-NPs

HSA is a protein that exhibits biocompatibility, biodegradability and high efficiency in drug binding. The ability to prepare nanoparticles from this polymer is relatively quick and simple [34]. Considering the analysis of 10-Pyr-3,6-DAPT interactions with HSA, it was decided to use this protein as a drug nanocarrier. Desolvation method is one of the most popular methods for nanoparticle synthesis. This is due to the possibility of relatively fast sample preparation and efficient encapsulation efficiency (EE) [34]. In the studies conducted, the encapsulation efficiency of 10-Pyr-3,6-DAPT in HSA nanoparticles is 99.65 ± 0.05%. Teran-Saavedra et al. [35] synthesized doxorubicin-loaded albumin/lactosylated (core-shell) nanoparticles (tBSA/BSALac NPs) with EE value equal to 91.00 ± 2.00%, where binding constant K of doxorubicin (DOX) with bovine serum albumin (BSA) at physiological conditions was 7.8 (±0.7) × 10^3^ M^−1^. Drug loading efficiency of entrapped DOX into HSA nanoparticles synthesized by Zhang et al. [20] was 85.8% for the molar ratio [HSA]:[DOX] 1:10. The K value of binding DOX with HSA equaled to 1.1 (±0.3) × 10^4^ M^−1^ [36]. The K of high-affinity binding sites for DOX bound to BSA hydrogel was calculated to be 1.6 × 10^5^ M^−1^ [37]. Molecular docking study of DOX binding to HSA has shown that DOX partially penetrates the Sudlow site I and can be localized in hydrophilic cleft formed by subdomains IIA and IIIA [38]. When analyzing the binding constant of chlorambucil to BSA, the value obtained was 5.58 × 10^4^ M^−1^,and the encapsulation efficiency of chlorambucil into BSA nanoparticles was different depending on the encapsulated chlorambucil concentration (EE from 90.08 ± 0.86% to 99.68 ± 0.06%) [20,35]. Moreover, chlorambucil binds to both Site I and Site II of BSA; however, Site II was predicted to be the preferred binding site [39]. New phenothiazine derivatives are not common compounds encapsulated in nanoparticles, but our previous work provides information on BSA nanoparticles with encapsulated 10*H*-2,7-diazafenothiazine, which EE was 66.67 ± 6.11% [21]. Based on the concluded study, the main binding site of 10-Pyr-3,6-DAPT is Sudlow site II, similar to the chlorambucil binding to BSA. When considering the results of encapsulation efficiency, it is important to note the method of nanoparticle preparation and the affinity of the drug for the polymer. However, the EE value is probably not affected by the exact albumin binding site bound by the drug.

### 3.3. Study of HSA and HSA-NPs Secondary Structure

Circular dichroism (CD) spectroscopy is a spectropolarimetric method that allows for studying the chiral molecules and secondary structure of proteins. α-helical structures have two negative bands at 222 nm and 208 nm, whereas the one positive band is about 193 nm [40]. In the present study, the interaction of HSA with 10-Pyr-3,6-DAPT and the changes in HSA secondary structure affected by the nanoparticle preparation process have been studied. As presented in Figure 6a, HSA is an α-helical protein with two characterized bands at approx. λ_min_ 210 nm and λ_min_ 220 nm. The presence of 10-Pyr-3,6-DAPT results in a change in the appearance of the spectrum, which is probably due to a change in the α-helical structure of the HSA or a small but significant ability of the substance to induce rotation of polarized light, so that a detection of λ_min_ for wavelengths in the 208–210 nm range was not possible. In our previous studies, no similar effect was observed [14,15,16]. In terms of nanoparticle preparation, desolvation process has a significant impact on the secondary structure of albumin, which can be seen in Figure 6b and is evidenced in our previous studies [40,41]. The presence of encapsulated 10-Pyr-3,6-DAPT is likely to have a protective effect on the secondary structure of albumin during the nanoparticle preparation. CD is not widely used in terms of nanoparticle study; however, it can provide information on the fate of the protein during nanocarrier preparation [40,41,42,43].

## 4. Materials and Methods

### 4.1. Study of HSA as a Carrier of 10-Pyr-3,6-DAPT

#### 4.1.1. Sample Preparation

A sample of 10-(2′-Pyrimidyl)-3,6-diazafenothiazine was obtained and purified according to the procedure described in the article [12], and its purity was confirmed by the melting point.

Based on the previously developed protocols [14,15,16], the HSA solution at 1 × 10^−6^ mol·L^−1^ concentrations was prepared in phosphate buffer (pH 7.4, 0.05 mol·L^−1^). A stock solution of 10-(2′-pyrimidyl)-3,6-diazafenothiazine (10-Pyr-3,6-DAPT) was prepared in dimethyl sulfoxide (DMSO), while dansyl-l-glutamine (dGlu) and dansyl-l-proline (dPro) solutions at 1 × 10^−3^ mol·L^−1^ concentration were prepared in methanol.

Ligand-protein binding interaction has been conducted at [10-Pyr-3,6-DAPT]:[HSA] 0:1–10:1 molar ratio. For the binding sites assessments, HSA in the absence and in the presence of fluorescent probes at [HSA]:[dGlu] 1:1 and [HSA]:[dPro] 1:1 molar ratios were titrated by 10-Pyr-3,6-DAPT from 1 × 10^−6^ mol·L^−1^ to 1 × 10^−5^ mol·L^−1^ concentrations.

#### 4.1.2. Steady State Fluorescence Measurements

All fluorescence measurements have been conducted at 298 K using fluorescence spectrophotometer JASCO FP-6500 (Hachioji, Japan) with quartz cells at 10 mm path length. Accuracy of wavelength was ± 1.5 nm. Steady state fluorescence spectra of HSA in the absence and the presence of 10-Pyr-3,6-DAPT at increasing concentration have been recorded using λ_ex_ 275 nm and λ_ex_ 295 nm excitation wavelengths. For the binding site assessment, the fluorescent probes [HSA]:[dGlu] and [HSA]:[dPro] 1:1 molar ratios were titrated by 10-Pyr-3,6-DAPT at 1 × 10^−6^ mol·L^−1^–1 × 10^−5^ mol·L^−1^ concentration using λ_ex_ 350 nm excitation wavelength. The scattering spectrum of solvent has been subtracted from all of the obtained spectra.

Due to the absorption of light at both excitation and emission wavelength (inner filter effect, IFE), a correction of 10-Pyr-3,6-DAPT-HSA system fluorescence intensity is required. Using a JASCO V-530 spectrophotometer, the absorbance measurements at the wavelength used to excite fluorophores fluorescence were made. For the inner filter correction equation, Equation (1) has been used [22]. This equation can be used as long as the absorbance increase of the system is not greater than ≈0.3:

(1) $$F_{cor}=F_{obs}\cdot e^{\frac{A_{ex}+A_{em}}{2}}$$

where

F_cor_ and F_obs_—corrected and observed fluorescence, respectively

A_ex_ and A_em_—absorbance at excitation and emission wavelength, respectively.

Spectral parameter A and full width at half maximum (FWHM) were used to assess changes in the environment of aromatic amino acids residues. Spectral parameter A was calculated based on Equation (2) [24]. In turn, FWHM was calculated based on the software provided by the manufacturer (Spectra Manager, version 2.13.00 2002–2015 Jasco Corporation):

(2) $$ParameterA={\left(\frac{F_{365}}{F_{320}}\right)}_{cor}$$

where

F_365nm_ and F_320nm_—the fluorescence intensity at λ 365 nm and 320 nm, respectively.

The fluorescence quenching effect (static and/or dynamic) of studied 10-Pyr-3,6-DAPT-HSA system have been analyzed according to the Stern-Volmer equation (Equation (3) [26]:

(3) $${\left(\frac{F_{0}}{F}\right)}_{cor}=1+k_{q}\cdot \tau_{0}\cdot \left[L\right]=1+K\cdot [L]$$

where

F and F_0_—the fluorescence intensity at the maximum wavelength of protein in the presence and absence of a quencher, respectively.

$k_{q}=\frac{K_{S-V}}{\tau_{0}}$—bimolecular quenching rate constant [mol^−1^·L·s^−1^].

$\tau_{0}$—the average fluorescence lifetime of protein without quencher ($\tau_{0(HSA)}$ = 6.000 × 10^−9^ s) [20].

[L]—ligand concentration [mol·L^−1^] ([L] = [L_b_] + [L_f_], where [L_b_] and [L_f_] are the bound and unbound (free) drug concentrations, respectively.

K_S-V—_Stern-Volmer constant [mol^−1^·L].

The association constants (K_a_) in 10-Pyr-3,6-DAPT-HSA complex was estimated using the Klotz equation (Equation (4)) [44]:

(4) $$\frac{1}{r}=\frac{1}{n}+\frac{1}{\left(n\cdot K_{a}\cdot [L_{f}]\right)}$$

r—number of ligand moles bound to 1 mole of protein $r=\frac{\left[L_{b}\right]}{\left[P\right]}$;

n—number of binding sites classes;

K_a_—association constant [mol^−1^·L];

[L_f_]—free ligand concentration [mol·L^−1^].

To determine the 10-Pyr-3,6-DAPT high affinity binding sites in HSA molecules, the percentage of fluorescent marker displacement (PD) from its binding site, i.e., dGlu and dPro from the HSA molecule, has been calculated utilizing Equation (5):

(5) $$PD={\left(\frac{F_{0}-F}{F_{0}}\right)}_{cor}\cdot 100\%$$

where

F and F_0_—the fluorescence intensity at the maximum wavelength of protein in the absence and presence of a quencher, respectively.

### 4.2. Study of 10-Pyr-3,6-DAPT Encapsulation into HSA-NPs

HSA nanoparticles with 10-Pyr-3,6-DAPT (10-Pyr-3,6-DAPT-HSA-NPs) were prepared using the desolvation method, according to procedures described before [34,40] with some modifications. HSA was dissolved in phosphate buffer, and 10-Pyr-3,6-DAPT was dissolved in methanol. Both were mixed together at [HSA]:[10-Pyr-3,6-DAPT] 1 × 10^5^:1 molar ratio. Ethanol was added dropwise into the mixture, to reach total volume of 10 mL. To achieve cross-linked nanoparticles, 21 µL of 8% aqueous glutaraldehyde solution was added. The process was performed for 20 h with magnetic stirring at 293.15 K in complete darkness. Nanoparticles without 10-Pyr-3,6-DAPT (HSA-NPs) were prepared according to the mentioned procedure but without drug added.

The suspension was purified by centrifugation in distilled water (293 K, 12,000 rpm), then was redispersed by vortex and ultrasonication. The pellet of nanoparticles was freeze-dried for further analysis, and supernatant containing unbound drug was collected for UV-Vis analysis immediately after the centrifugation process. In order to determine the encapsulation efficiency (EE), Equation (6) was used:

(6) $$EE=\frac{drug\;added\;\left(mg\right)-free\;drug\;\left(mg\right)\;}{drug\;added\;(mg)}\cdot 100\%$$

The obtained nanoparticle solutions remained stable during centrifuge, vortex and ultrasonication.

### 4.3. Study of HSA and HSA-NPs Secondary Structure

Far UV-CD spectra of HSA and 10-Pyr-3,6-DAPT-HSA complexes at [HSA]:[10-Pyr-3,6-DAPT] 1:4 molar ratio, as well as HSA-NPs and 10-Pyr-3,6-DAPT-HSA-NPs, both at protein concentration 0.5 × 10^−6^ [mol·L^−1^], were recorded using a JASCO J-1500 spectropolarimeter (Hachioji, Japan). The measurements were conducted in nitrogen atmosphere at 293.15 K in quartz cuvettes with an optical path of 1 mm. The spectra were recorded in the wavelength range from 190 to 260 nm at wavelength intervals of 0.2 nm. The accuracy of the wavelength measurement was ±0.1 nm, and the wavelength repeatability was ±0.05 nm. CD protein of human serum albumin in the absence and in the presence of 10-Pyr-3,6-DAPT as well as in nanoparticles after the preparation process was corrected by subtraction of spectra obtained for phosphate buffer at pH 7.4 and smoothed using Savitzky and Golay filters method [39].

## 5. Conclusions

The main aim of the study was to analyze both human serum albumin (HSA) and albumin nanoparticles (HSA-NPs) as carriers of 10-(2′-pyrimidyl)-3,6-diazaphenothiazine (10-Pyr-3,6-DAPT) using spectrofluorescence, UV-Vis and circular dichroism (CD) spectroscopy. Based on the conducted studies, the 10-Pyr-3,6-DAPT probably binds in one class of HSA binding site, and the binding has been localized in Sudlow site II. Moreover, 10-Pyr-3,6-DAPT modifies the tertiary structure of HSA as well as its secondary structure. The high value of encapsulation efficiency suggests that albumin nanoparticles prepared with desolvation method could be a potential formulation of 10-Pyr-3,6-DAPT delivery system. In terms of CD analysis, the substance used probably exerting a protective effect on the secondary structure of HSA during nanocarrier preparation. Until now, there have been few studies concerning the in vitro spectroscopic analysis of HSA and HSA-NPs as carriers of 10-Pyr-3,6-DAPT. The conducted studies are basic; however, they shed light on the interaction of the potential drug with the most common plasma protein and proposed its form of delivery in a biocompatible biopolymer, which is a valuable contribution from a scientific point of view. Nevertheless, further in vitro and in vivo studies are needed to thoroughly investigate the potential of this substance.

## Figures and Tables

**Table 1:** Spectral parameter A and FWHM at λ_ex_ 275 nm and λ_ex_ 295 nm excitation wavelengths.

[10-Pyr-3,6-DAPT]:[HSA] Molar Ratio	λ_ex_ 275 nm			λ_ex_ 295 nm		
	λ_max_ [nm]	Parameter A ±SD ^1^	FWHM [nm] ±SD ^1^	λ_max_ [nm]	Parameter A ±SD ^1^	FWHM [nm] ±SD ^1^
0:1	329	0.71 ± 0.01	64.87 ± 0.41	336	0.99 ± 0.04	52.43 ± 0.12
10:1	324	0.70 ± 0.03	62.14 ± 0.23	335	0.86 ± 0.02	52.33 ± 0.09

^1^ SD—standard deviation.

**Table 2:** The percentage of HSA (1 × 10^−6^ mol·L^−1^) fluorescence quenching in presence of 10-Pyr-3,6-DAPT (1 × 10^−6^ mol·L^−1^–1 × 10^−5^ mol·L^−1^) at λ_ex_ 275 nm and λ_ex_ 295 nm excitation wavelengths.

[10-Pyr-3,6-DAPT]:[HSA] Molar Ratio	Percentage [%] of HSA Fluorescence Quenching ±SD ^1^	
	λ_ex_ 275 nm	λ_ex_ 295 nm
10:1	15.33 ± 1.13	10.65 ± 2.34

^1^ SD—standard deviation.

**Table 3:** Stern-Volmer constant (K_S-V_) and bimolecular quenching constant rate (k_q_) for 10-Pyr-3,6-DAPT-HSA complex at λ_ex_ 275 nm and λ_ex_ 295 nm excitation wavelengths; [10-Pyr-3,6-DAPT]:[HSA] 0:1–10:1 molar ratio.

[10-Pyr-3,6-DAPT]:[HSA] Molar Ratio	λ_ex_ 275 nm		λ_ex_ 295 nm	
	K_S-V_ ± SD ^1^ [mol^−1^·L]	k_q_ ± SD ^1^ [mol^−1^·L·s^−1^]	K_S-V_ ± SD ^1^ [mol^−1^·L]	k_q_ ± SD ^1^ [mol^−1^·L·s^−1^]
10:1	1.89 × 10^4^ ± 0.09	3.16 × 10^12^ ± 0.09	1.22 × 10^4^ ± 0.04	2.04 × 10^12^ ± 0.07

^1^ SD—standard deviation.

**Table 4:** Binding parameters for 10-Pyr-3,6-DAPT-HSA complex at λ_ex_ 275 nm and λ_ex_ 295 nm excitation wavelengths; [10-Pyr-3,6-DAPT]:[HSA] 0:1–10:1 molar ratio.

	λ_ex_ 275 nm		λ_ex_ 275 nm	
[10-Pyr-3,6-DAPT]:[HSA] Molar Ratio	K_a_ [mol^−1^·L] ± SD ^1^	n ± SD ^1^	K_a_ [mol^−1^·L] ± SD^1^	n ± SD ^1^
10:1	(5.24 ± 0.57) × 10^4^	0.91 ± 0.13	(4.67 ± 0.59) × 10^4^	1.03 ± 0.17

^1^ SD—standard deviation.

**Table 5:** The percentage of dGlu and dPro displacement from HSA high affinity binding site; [HSA]:[dGlu] = [HSA]:[dPro] 1:1 molar ratio.

C_10-Pyr-3,6-DAPT_ [mol·L^−1^]	[HSA]:[dGlu] Molar Ratio	[HSA]:[dPro] Molar Ratio
	1:1	
	Percentage [%] of Displacement ± SD ^1^	
0	-	-
1 × 10^−5^	39.86 ± 1.09	56.21 ± 0.56

^1^ SD—standard deviation.

## Data Availability

Data is contained within the article.
